# 230 days of ultra long‐term subcutaneous EEG: seizure cycle analysis and comparison to patient diary

**DOI:** 10.1002/acn3.51261

**Published:** 2020-12-04

**Authors:** Pedro F. Viana, Jonas Duun‐Henriksen, Martin Glasstëter, Matthias Dümpelmann, Ewan S. Nurse, Isabel P. Martins, Sonya B. Dumanis, Andreas Schulze‐Bonhage, Dean R. Freestone, Benjamin H. Brinkmann, Mark P. Richardson

**Affiliations:** ^1^ Institute of Psychiatry, Psychology and Neuroscience King’s College London London United Kingdom; ^2^ Faculty of Medicine University of Lisbon Lisboa Portugal; ^3^ UNEEG medical A/S Lynge Denmark; ^4^ Epilepsy Center Department for Neurosurgery University Medical Center Freiburg Freiburg Germany; ^5^ Seer Medical Inc. Melbourne Victoria Australia; ^6^ Department of Medicine St. Vincent’s Hospital University of Melbourne Melbourne Victoria Australia; ^7^ Epilepsy Foundation Landover Maryland USA; ^8^ Mayo Systems Electrophysiology Laboratory Department of Neurology Department of Physiology and Biomedical Engineering Mayo Clinic Rochester Minnesota USA

## Abstract

We describe the longest period of subcutaneous EEG (sqEEG) monitoring to date, in a 35‐year‐old female with refractory epilepsy. Over 230 days, 4791/5520 h of sqEEG were recorded (86%, mean 20.8 [IQR 3.9] hours/day). Using an electronic diary, the patient reported 22 seizures, while automatically‐assisted visual sqEEG review detected 32 seizures. There was substantial agreement between days of reported and recorded seizures (Cohen’s kappa 0.664), although multiple clustered seizures remained undocumented. Circular statistics identified significant sqEEG seizure cycles at circadian (24‐hour) and multidien (5‐day) timescales. Electrographic seizure monitoring and analysis of long‐term seizure cycles are possible with this neurophysiological tool.

## Introduction

For patients with refractory epilepsy, repetitive seizure occurrence presents significant challenges adding to overall disease burden. Unpredictability of seizures, coupled with the potential for seizure‐related injury or death, prompts patients to adopt longstanding lifestyle limitations affecting their independence and quality of life.^1^ A second challenge is accurate seizure documentation. Seizure diaries, the current standard assessment of seizure occurrence at home, have been shown to be unreliable with a tendency to under‐report events or to misattribute certain events as seizures, leading to inappropriate treatment management.^2^, ^3^

There is an ongoing search for technological solutions to provide remote, continuous and long‐term measurements of biosignals related to seizure occurrence or propensity. Key requirements for these devices are that they are reliable and acceptable by users.^4^ Currently marketed seizure detection devices are restricted to the detection of convulsive seizures.^4^

EEG is the hallmark of seizure detection of any type. Multiple seizure detection algorithms have been developed for scalp EEG,^5^ although scalp electrodes are not technically feasible for long‐term recordings. Conversely, long‐term intracranial EEG systems have technical limitations and require major surgery susceptible to serious post‐operative complications.^3^, ^6^, ^7^ Minimally invasive subcutaneous EEG (sqEEG) could provide a balance between good quality data and patient usability/acceptability. sqEEG recordings show similar objective^8^ and subjective^9^ signal quality compared to scalp EEG. Recently, a 9‐patient trial of sqEEG monitoring for up to 92 days demonstrated overall good safety and high adherence rate.^10^

We report the longest trial of sqEEG to date (>7 months), focusing on usability, comparison to the patient’s diary and the analysis of long‐term seizure risk cycles, known to improve personalized seizure forecasts.^11^, ^12^, ^13^

## Case Report

A 35‐year‐old woman presented with focal epilepsy since age 28. She described three major seizure types: (1) focal aware (FAS), with *déjà‐vù*, intense anxiety, nonrising epigastric sensation, and involuntary jaw clenching, all lasting 30 sec; (2) focal impaired awareness (FIAS), with sudden loss of awareness and jaw clenching for 2–3 min, occasional urinary incontinence and tongue biting; (3) focal to bilateral tonic‐clonic (FBTCS), evolving from FAS/FIAS. Seizure frequency was 3‐4 FAS every 6 weeks and one FIAS per month while no FBTCS were reported in the last two years. FIAS were frequently unnoticed by the patient unless witnessed or preceded by a FAS.

On presurgical investigation, brain MRI was compatible with a right amygdala/hippocampus Dysembrioplastic Neuroepithelial Tumor (Figure S1). Inpatient scalp video‐EEG with antiepileptic drug reduction showed bilateral independent temporal interictal discharges (more on the left), and multiple FIAS were recorded showing prominent muscle artefact (jaw clenching), with evolving theta‐delta activity followed by post‐ictal slowing in the left temporal region (Figures S2 and S3). Intracranial video‐EEG showed bilateral independent‐onset mesial temporal seizures; while some subclinical seizures remained restricted to the right mesial temporal region, all clinical seizures had a left temporal electrographic correlate (onset or spread). Resective surgery was not indicated.

The patient was enrolled in an observational seizure forecasting study (clinicaltrials.gov NCT04061707), approved by the local ethics committee (19/LO/0354). Written informed consent was obtained. The sqEEG device (UNEEG SubQ™) consists of a 3‐contact electrode (yielding 2‐channel bipolar EEG) and a small housing, implanted unilaterally under local anesthesia, over the region of pre‐identified ictal EEG changes.^8^ An external recorder (24/7 EEG™ SubQ) connects to the implant housing via an inductive link, powering the implant and recording data (sampling rate: 207Hz).

The device was implanted over the left temporal region (after consensus discussion based on the previous investigation). The patient reported moderate headache post‐implantation, gradually subsiding over three weeks; no other clinical adverse events were reported. Starting two weeks after implantation, the subject was instructed to record for as long as possible, while also reporting her seizures as accurately as possible on an electronic diary (Seer App). Retrospective logging of seizures was also allowed. Monthly visits were undertaken for data collection and diary review. No changes in usual medication occurred throughout the study (levetiracetam 1500mg and carbamazepine 600mg, both twice‐daily).

**Figure 1:**
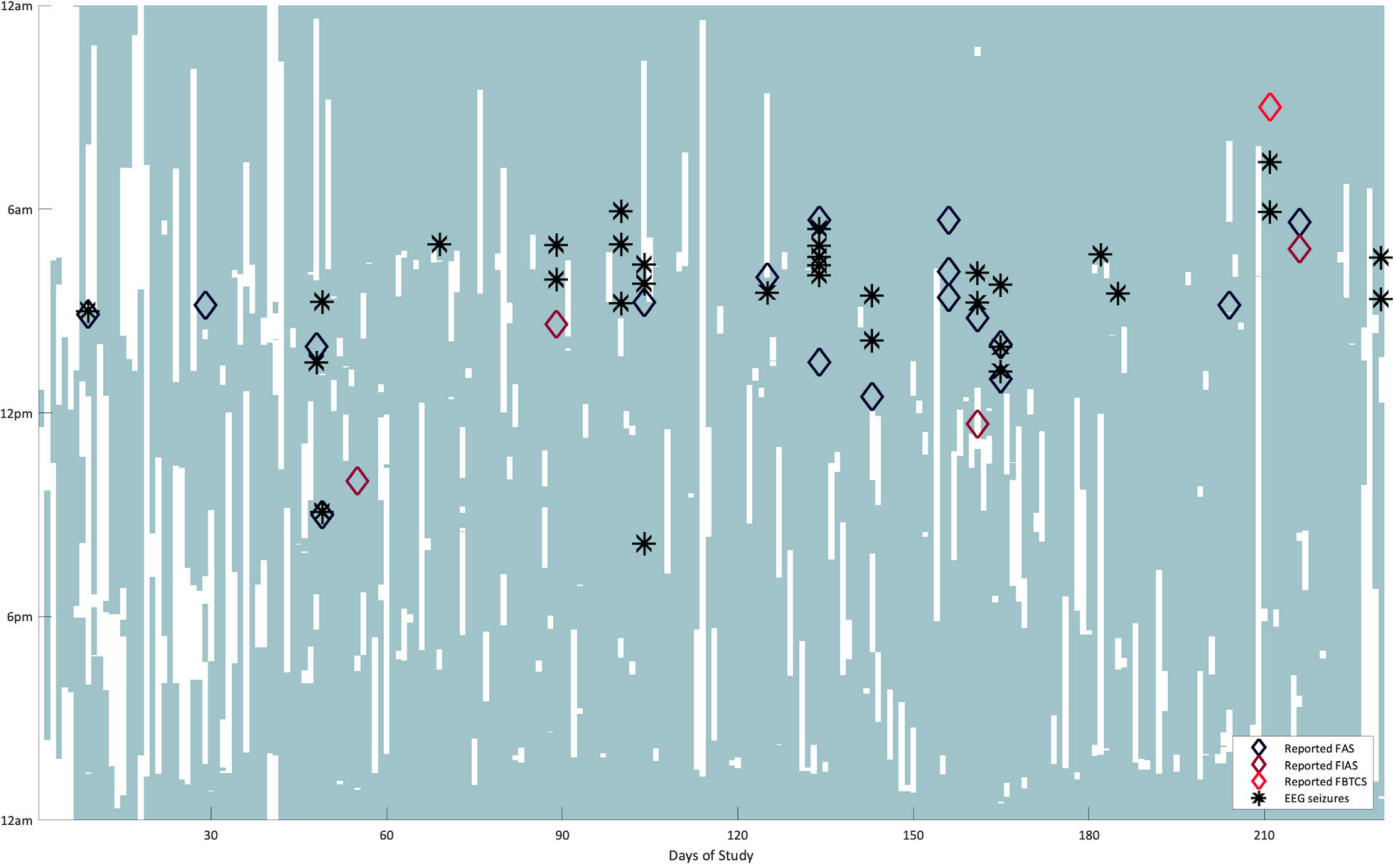
Day of study (x‐axis) versus time of day (y‐axis) plot, showing recording time with the sqEEG system (shaded in light green), with superimposed times of EEG seizures (black asterisks) and of reported events in the electronic diary (diamonds).

Data collection of sqEEG was prematurely interrupted after 230 days due to a device malfunction. The manufacturer analyzed the root cause and updated the design to obtain a more robust device. Over the study period, the patient recorded a total of 4791.2 h (86.8% adherence, mean 20.8 h/day). Recording time improved over the first two months, then remained stable throughout the study (Figure 1). Reasons for device disconnection, besides personal hygiene, were accidental disconnections at night, when exercising, or malfunction of the recorder. Total device deficiency time, including time whilst not recording and time during device malfunction, was 849.1h (15.4% of the study period).

Electrographic seizures were identified offline by visual review of the timeseries on dedicated software (UNEEG™ Episight) by a reviewer (PFV) experienced in sqEEG. We reviewed epochs identified by a high sensitivity scalp‐EEG automated seizure detector, together with a random sample of 6‐hour epochs comprising 10% of the whole recording, and for +/‐ 3h around diary events. Taken together, approximately 14% of the recording (669 h) was reviewed. Previous ictal scalp video‐EEG was used as a visual guide (seizure signature). A 15‐minute EEG quality protocol, performed with the patient at two separate visits, recorded common physiological artefacts such as chewing and jaw clenching that also served as (negative) visual guides.

Throughout the study, the patient reported 22 seizures, mostly FAS (*n* = 17), four FIAS and one suspected FBTCS (having woken up in the morning with intense headache and bitten cheek). After sqEEG visual review, 32 seizures were identified, all with similar electrographic characteristics to the patient’s scalp video‐EEG (Figure S3). All were identified by the seizure detector, and all had clinical manifestations evident by the presence of EMG artefact reflecting jaw clenching. The first seizure occurred at day 15 of the study. Two seizures were recorded during the night of the reported “FBTCS”, neither of which showed tonic‐clonic artefact (i.e. both consistent with FIAS). One reported FAS coincidentally occurred during a study visit, with an ictal electrographic correlate seen on visual review. The number of recorded seizures increased from the first three months (mean 2.3 seizures/month) to the remaining study period (mean 5.6 seizures/month). Seizures frequently clustered throughout the day. There was substantial agreement between days of reported seizures and of sqEEG seizures (Cohen’s κ: 0.664). However, after matching individual sqEEG seizures and reported diary events according to their temporal vicinity (+/‐ 1h), only 9 sqEEG seizures were associated with a reported event (F1 agreement score: 0.33). In parallel, 13 reported events were not timed to a sqEEG seizure, with missing recording periods on three occasions. Longer duration sqEEG seizures tended to be more frequently reported than shorter ones (medians 84.2s vs. 62.9s, Mann–Whitney U‐test *P* = 0.05).

**Figure 2:**
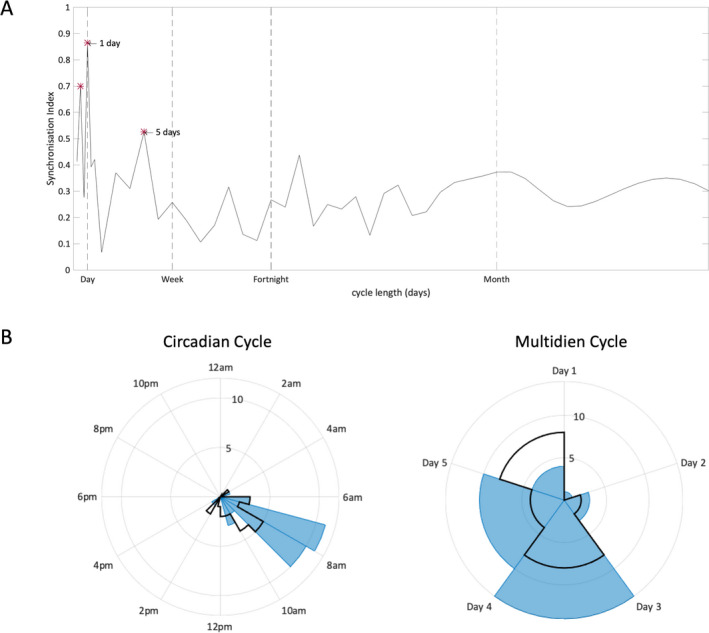
(A) Distribution of the synchronization index (SI) of sqEEG seizures at different cycle lengths. The SI value reflects the degree of alignment of seizures to a particular phase of each cycle, when plotted on a circular graph, and ranges from 0 (randomly distributed events) to 1 (completely aligned events).^13^ Significant cycles were assessed with the Rayleigh’s test for non‐uniformity (*P* < 0.05 with Bonferroni correction for the number of studied cycles) and are highlighted with an asterisk. Note the harmonic cycle at 12 h (not visible in the circadian polar histogram below). (B) Polar histograms of sqEEG seizures (blue) and diary events (black outline) distributed over the significant sqEEG seizure cycles (24h and 5 days). Concentric rings represent number of events/seizures.

Analysing the distribution of seizures within a range of temporal cycles, two significant sqEEG seizure cycles were identified from a range of potential cycle lengths (significance assessed by the Rayleigh’s test for circular nonuniformity, corrected for the number of studied cycles).^12^, ^14^ One very strong circadian cycle was demonstrated at 24h with seizures clustered in the early morning (7‐9am), and a weaker long‐term cycle was seen at 5 days (Figure 2). Only the 24‐hour cycle was detected based on the diary data alone, with a tendency for the patient to report seizures later in the day.

## Discussion

We successfully recorded ultra‐long‐term (>7 months) sqEEG in a single patient, in an ambulatory, real‐life setting. After a short adaptation period, the system was well accepted, and no serious adverse events were reported. We show similarly high average adherence to the previous three‐month trial using the same system, but for a longer period of time.^10^ This is in contrast with concerns of high attrition rate when using wearable devices in healthcare settings,^15^ reflecting the motivation of (at least some) patients with epilepsy towards long‐term at‐home recordings.

All EEG‐detected seizures were associated with clinical manifestations, as muscle artefact (similarly seen during the patient’s video‐EEG recorded seizures) was co‐occurrent with EEG changes, and are hence suspected to be clinically relevant. Comparing diary events to sqEEG seizures, it is clear that the patient is a good reporter, especially when considering the day of seizure occurrence (and the morning propensity of seizure occurrence). Still, several undocumented sqEEG seizures were recorded, with less than a third of sqEEG seizures temporally coinciding with a reported event. Multiple subclinical FIAS (often occurring in clusters as seen in Figure 1) are a likely explanation, in agreement with previous studies of inaccuracy of patient diaries.^2^, ^3^ Peri‐ictal impaired perception and/or forgetfulness to document perceived seizures, especially when coupled to retrospective logging of events, may be reasons for this innacuracy.^2^ Our patient’s tendency to better report longer seizures may suggest these to be clinically more severe, or long enough to be noticed by the patient or witnesses.

We cannot exclude that seizures could have occurred outside periods of active recording. However, only a minority of diary events (*n* = 3) were reported at times with missing recording data. Moreover, FAS,

often not associated with ictal EEG changes, could have escaped detection, although throughout monitoring several reported FAS were closely timed to sqEEG seizures (including a FAS occurring during a study visit). Due to limited (unilateral) spatial sampling and the history of bilateral temporal seizures, we also cannot exclude undetected contralateral seizures, which could possibly explain some of the patient‐reported events without sqEEG correlates. Reasons against this hypothesis include historical evidence from in‐hospital scalp video‐EEG, with all clinical right‐sided seizures including ictal and post‐ictal changes in the sampled left temporal region. It is possible, however, that medication withdrawal could be a factor in allowing wider/contralateral spread during video‐EEG, and seizures occurring in an ambulatory setting might not always spread to the sqEEG‐sampled region. Furthermore, the semiology of right and left temporal onset seizures identified by intracranial EEG was indistinguishable, although recorded seizures restricted to the right mesial temporal lobe were purely electrographic.

The patient reported one seizure as a FBTCS. However, on visual EEG review, the closely timed detected seizure did not show tonic‐clonic movement artefacts. Although requiring future validation with concurrent video recordings, this finding extends the potential use‐case scenarios of sqEEG monitoring. There are important implications in patient management to identifying TCS for individual estimation of SUDEP risk (highly associated tonic‐clonic seizures).^16^

In this patient, we identified two significant circadian and multidien cycles. The circadian periodicity with clustered morning seizures was very strong and also evident from the diary data, while the multidien 5‐day cycle was weaker with seizures distributed over a wider phase, and was not detectable in diary data (Figure 2). Given the high device recording adherence throughout, we do not believe that cycle analysis in this case was affected by periods of non‐recording. Such ultra‐long‐term recordings are essential to provide adequate statistical sampling power for characterization of seizure risk cycles and for seizure forecasting.^11^, ^13^, ^14^ Future work should investigate the clinical benefit of seizure forecasting from sqEEG, given its low invasiveness but also its challenges (i.e. missed seizures due to limited spatial sampling, including of deep cortical sources). The observed clustering of seizures is also notable here, and is an important consideration in seizure forecasting applications.

Visual review of sqEEG data presents a challenge in long‐duration studies. Experience with previous sqEEG datasets suggests the incorporation of visual guides for seizure identification (i.e. previous ictal scalp video‐EEG data) and for rejection of common artefacts (e. g. with artefact recording protocols) are certainly of value in increasing reviewer confidence. The high‐sensitivity seizure detector used most likely detected all seizures (after comparing with review of a proportion of the dataset) but the number of false positive events was high (*n* = 4768, ~1/h). Future studies should investigate the refinement in detection performance, in order to optimize visual review effort in ultra‐long‐term datasets.

In conclusion, we report a case of highly‐adherent, safe, minimally‐invasive ultra‐long‐term sqEEG monitoring. We demonstrate that sqEEG provides complementary information with high potential clinical value, calling for future investigation in larger cohort studies: detection of undocumented seizures, improved seizure timing, potential classification of major seizure types, and the recording of long‐term trends of seizure occurrence.

## Conflicts of Interest

JDH is an employee of UNEEG medical A/S. ESN and DF are employees and shareholders of Seer Medical. BHB has equity in Cadence Neurosciences. MPR has been a member of ad‐hoc advisory boards for UNEEG medical A/S. PFV received a payment from UNEEG medical A/S for data annotation in an unrelated research study. No other authors have conflicts to declare.

## Author Contributions

PFV, JDH, SD, ASB, DF, BHB, and MPR contributed to study conception. PFV, JDH, MD, MG, EN, IPM, ASB, DF, BHB, and MPR contributed to study design. PFV, JDH, and EN contributed to data collection. PFV, JDH, MG, MD, IPM, and MPR contributed to data analysis. PFV contributed to manuscript draft. All authors contributed to manuscript editing.

## Supporting information


**Figure S1.** T2 Axial Brain MRI slices showing lesion composed of a cluster of small cysts within the right amygdala, with no mass effect and no pathological enhancement, compatible with a dysembrioplastic neuroepithelial tumor (DNET).
**Figure S2.** Example of a seizure recorded during scalp video‐EEG. A theta‐delta evolving pattern is seen over the left hemisphere. Sampling rate 256Hz, transverse montage, bandpass filtered at [0.5 20] Hz.
**Figure S3.** Seizure examples from both inpatient scalp video‐EEG (top left, large panel, left frontotemporal channels) and sqEEG (small panels), showing both time series and time‐frequency representation of each channel data. Time‐frequency decomposition was constructed via complex Morlet wavelet convolution, with wavelet frequencies between 0.5 and 40Hz and number of cycles between 5 and 20, both logarithmically spaced. As shown, seizures from both recording techniques are characterized by characteristic muscle artefact due to jaw clenching, and an underlying theta‐delta pattern, followed by post‐ictal delta activity. Some seizure segments contain signal artefacts (characterized by repetitive high amplitude spikes alternating with flat signal) due to electrode malfunction (f.eg. seizure 10).
