# Supplementary material for: 230 days of ultra long‐term subcutaneous EEG: seizure cycle analysis and comparison to patient diary
Source: Ann Clin Transl Neurol. 2020 Dec 4;8(1):288–93. doi: 10.1002/acn3.51261 (PMC7818131; doi:10.1002/acn3.51261)
Supplement: Supplementary file 1 — Figure S1. T2 Axial Brain MRI slices showing lesion composed of a cluster of small cysts within the right amygdala, with no mass effect and no pathological enhancement, compatible with a dysembrioplastic neuroepithelial tumor (DNET). Figure S2. Example of a seizure recorded during scalp video‐EEG. A theta‐delta evolving pattern is seen over the left hemisphere. Sampling rate 256Hz, transverse montage, bandpass filtered at [0.5 20] Hz. Figure S3. Seizure examples from both inpatient scalp video‐EEG (top left, large panel, left frontotemporal channels) and sqEEG (small panels), showing both time series and time‐frequency representation of each channel data. Time‐frequency decomposition was constructed via complex Morlet wavelet convolution, with wavelet frequencies between 0.5 and 40Hz and number of cycles between 5 and 20, both logarithmically spaced. As shown, seizures from both recording techniques are characterized by characteristic muscle artefact due to jaw clenching, and an underlying theta‐delta pattern, followed by post‐ictal delta activity. Some seizure segments contain signal artefacts (characterized by repetitive high amplitude spikes alternating with flat signal) due to electrode malfunction (f.eg. seizure 10). [file ACN3-8-288-s001.pdf]

Supplementary Figures

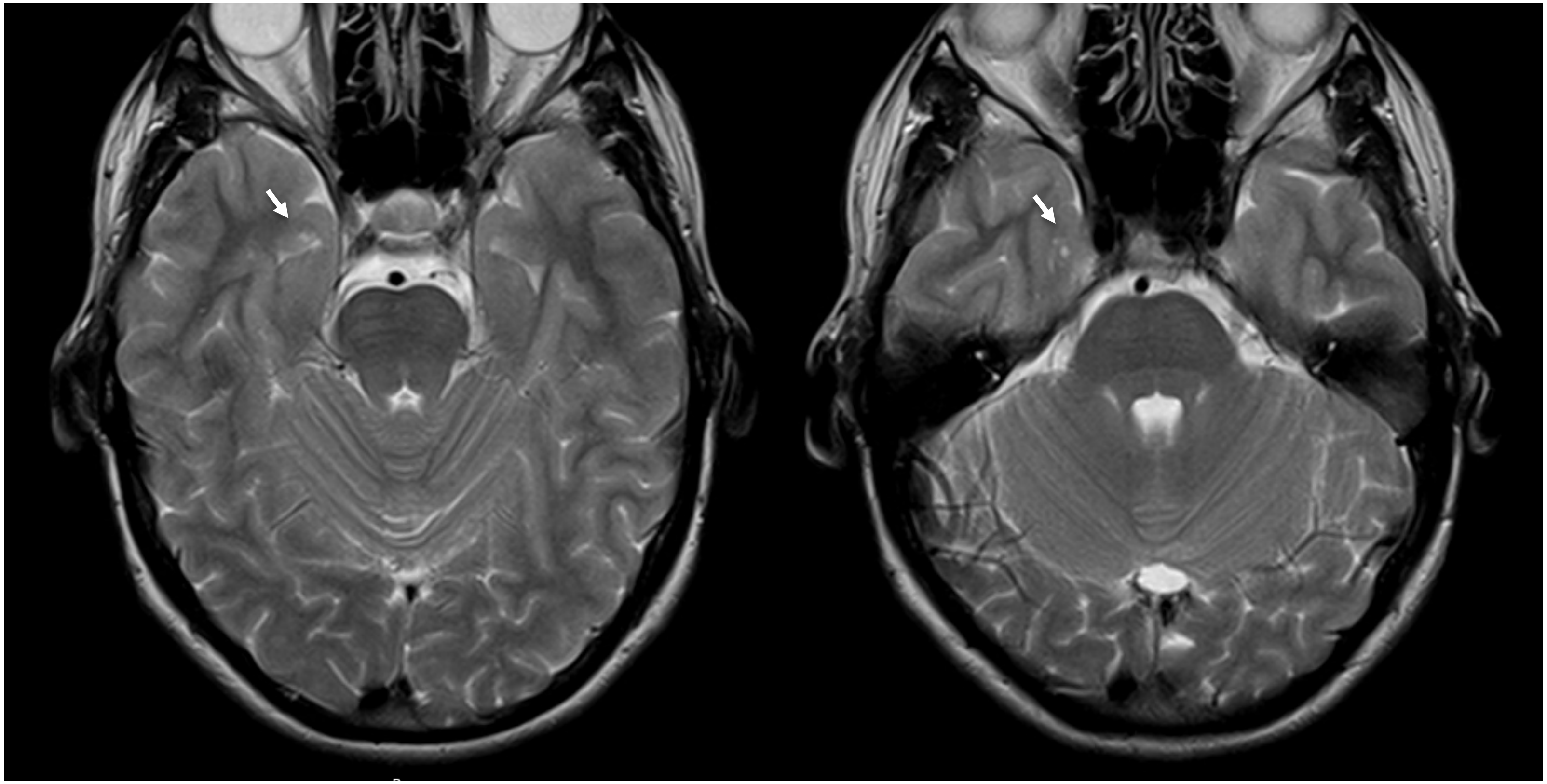

Supplementary Figure 1. T2 Axial Brain MRI slices showing lesion composed of a cluster of small cysts within the right amygdala, with no mass effect and no pathological enhancement, compatible with a dysembrioplastic neuroepithelial tumour (DNET).

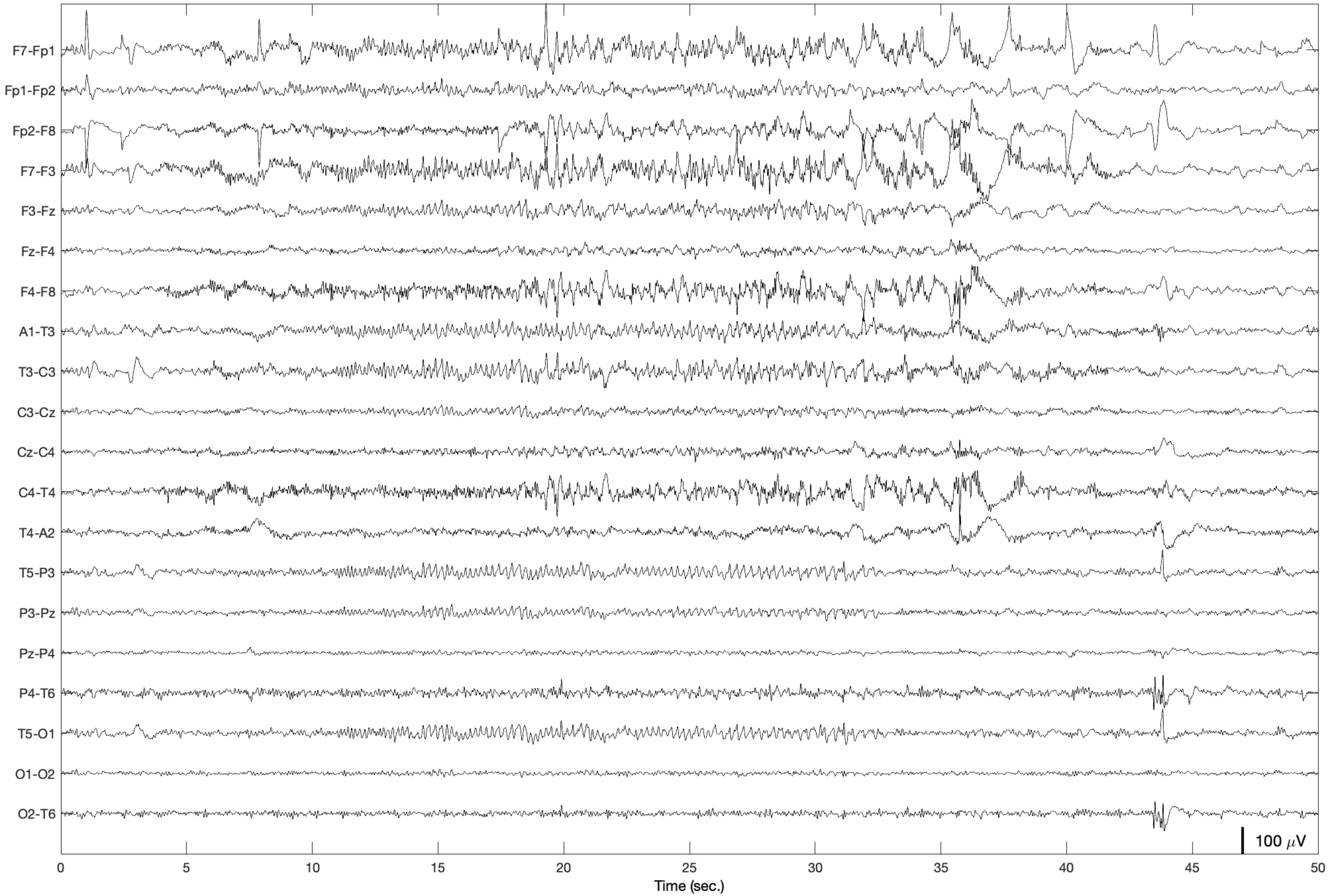

Supplementary Figure 2. Example of a seizure recorded during scalp video-EEG. A theta-delta evolving pattern is seen over the left hemisphere. Sampling rate 256Hz, transverse montage, bandpass filtered at [0.5 20] Hz.

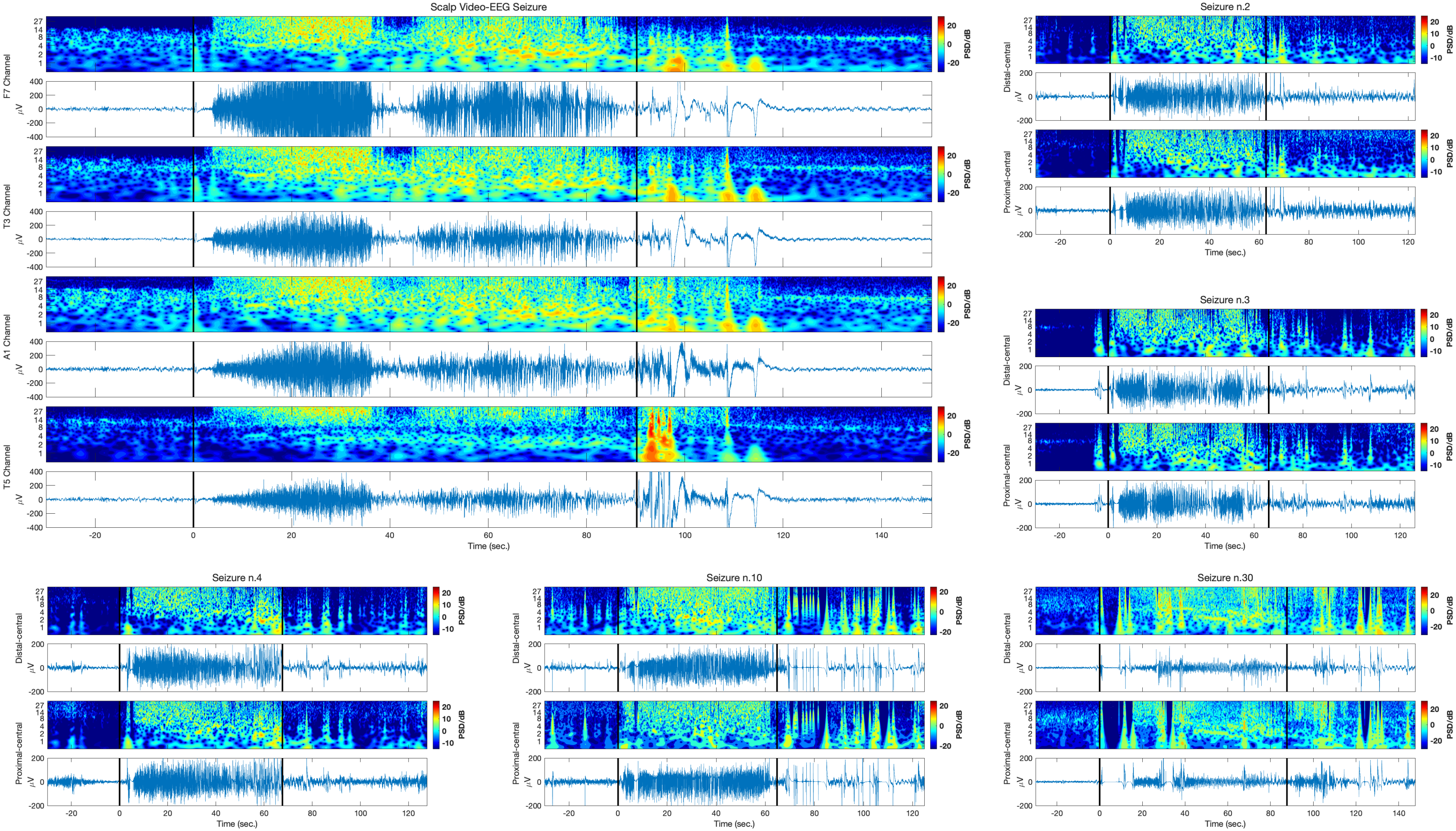

Supplementary Figure 3. Seizure examples from both inpatient scalp video-EEG (top left, large panel, left frontotemporal channels) and sqEEG (small panels), showing both time series and time-frequency representation of each channel data. Time-frequency decomposition was constructed via complex Morlet wavelet convolution, with wavelet frequencies between 0.5 and 40Hz and number of cycles between 5 and 20, both logarithmically spaced. As shown, seizures from both recording techniques are characterized by characteristic muscle artefact due to jaw clenching, and an underlying theta-delta pattern, followed by post-ictal delta activity. Some seizure segments contain signal artefacts (characterized by repetitive high amplitude spikes alternating with flat signal) due to electrode malfunction (f.eg. seizure 10).
